# Mucopolysaccharidosis IVA (Morquio A syndrome) and VI (Maroteaux–Lamy syndrome): under-recognized and challenging to diagnose

**DOI:** 10.1007/s00256-013-1797-y

**Published:** 2014-01-04

**Authors:** Ralph S. Lachman, Barbara K. Burton, Lorne A. Clarke, Scott Hoffinger, Shiro Ikegawa, Dong-Kyu Jin, Hiroki Kano, Ok-Hwa Kim, Christina Lampe, Nancy J. Mendelsohn, Renée Shediac, Pranoot Tanpaiboon, Klane K. White

**Affiliations:** 1 International Skeletal Dysplasia Registry, Cedars-Sinai Medical Center/University of California, Los Angeles, Los Angeles, CA USA; 2 Ann and Robert H. Lurie Children’s Hospital of Chicago and Northwestern University Feinberg School of Medicine, Chicago, IL USA; 3 Child and Family Research Institute, University of British Columbia, Vancouver, BC Canada; 4 Stanford University School of Medicine, Stanford, CA USA; 5 Laboratory for Bone and Joint Diseases, Centre for Integrative Medical Sciences, RIKEN, Tokyo, Japan; 6 Sungkyunkwan University School of Medicine, Seoul, South Korea; 7 Ajou University Hospital, Suwon, South Korea; 8 University Medical Center, University of Mainz, Mainz, Germany; 9 Children’s Hospitals and Clinics of Minnesota, Minneapolis, MN USA; 10 BioMarin Pharmaceutical Inc., Novato, CA USA; 11 Children’s National Medical Center, Washington, DC USA; 12 Seattle Children’s Hospital, Seattle, WA USA; 13 International Skeletal Dysplasia Registry, c/o 325 Channing Ave, #111, Palo Alto, CA 94301 USA

**Keywords:** Mucopolysaccharidosis, MPS, Morquio, Morquio A, Maroteaux-Lamy, MPS IVA, MPS VI, Dysostosis multiplex, Multiple epiphyseal dysplasia, MED, Spondylo-epiphyseal dysplasia, SED

## Abstract

**Objective:**

Mucopolysaccharidosis IVA (MPS IVA, or Morquio A syndrome) and VI (MPS VI, or Maroteaux–Lamy syndrome) are autosomal recessive lysosomal storage disorders. Skeletal abnormalities are common initial presenting symptoms and, when recognized early, may facilitate timely diagnosis and intervention, leading to improved patient outcomes. Patients with slowly progressing disease and nonclassic phenotypes can be particularly challenging to diagnose. The objective was to describe the radiographic features of patients with a delayed diagnosis of MPS IVA or VI.

**Materials and Methods:**

This was a retrospective study. The records of 5 MPS IVA and 3 MPS VI patients with delayed diagnosis were reviewed. Radiographs were evaluated by a radiologist with special expertise in skeletal dysplasias.

**Results:**

An important common theme in these cases was the appearance of multiple epiphyseal dysplasia (MED) with epiphyseal changes seemingly confined to the capital (proximal) femoral epiphyses. Very few patients had the skeletal features of classical dysostosis multiplex.

**Conclusions:**

Radiologists should appreciate the wide phenotypic variability of MPS IVA and VI. The cases presented here illustrate the importance of considering MPS in the differential diagnosis of certain skeletal dysplasias/disorders, including MED, some forms of spondylo-epiphyseal dysplasia (SED), and bilateral Perthes-like disease. It is important to combine radiographic findings with clinical information to facilitate early testing and accurate diagnosis.

## Introduction

The mucopolysaccharidoses (MPS) are lysosomal storage disorders caused by defects in glycosaminoglycan (GAG) catabolism [1] and are classified under the dysostosis multiplex group of skeletal dysplasias [2]. The recent emergence of specific therapies for some of the MPS disorders has brought to the forefront the importance of accurate and early diagnosis as a means of improving patient outcomes [3]. However, limited disease awareness and extensive clinical heterogeneity contribute to delays in the diagnosis of these rare disorders. Many radiographic and clinical manifestations of MPS may mimic those of other skeletal dysplasias, further complicating the path to diagnosis. Moreover, once clinical suspicion has been established, limitations and complications associated with diagnostic assays may delay definitive confirmation [4, 5].

In this paper, we present 8 cases that highlight the challenges associated with diagnosing MPS IVA (Morquio A syndrome) and MPS VI (Maroteaux–Lamy syndrome), two MPS disorders for which enzyme replacement therapy (ERT) is available or in development [6, 7]. MPS IVA is an autosomal recessive condition resulting from insufficient activity of *N*-acetylgalactosamine-6-sulfatase (GALNS), an enzyme that degrades keratan sulfate and chondroitin-6-sulfate [8]. MPS VI is an autosomal recessive disorder caused by deficient activity of *N*-acetylgalactosamine 4-sulfatase (arylsulfatase B or ARSB), the enzyme that catabolizes dermatan sulfate [9]. Classic signs and symptoms include short stature, skeletal abnormalities with some of the characteristic radiographic changes of dysostosis multiplex, joint problems, respiratory complications, cardiac disease, ocular abnormalities, and hearing loss [8–11]. The onset of symptoms usually occurs within the first 2 years of life in severely affected patients. In contrast, individuals with more slowly progressive disease typically develop symptoms later in life and may present with more subtle disease manifestations, increasing the likelihood of a delayed or missed diagnosis [12–15]. Many MPS IVA and VI individuals are at risk of multisystem impairment and significant morbidity, underscoring the importance of early diagnosis and multidisciplinary management, regardless of phenotype [8, 10, 16, 17]. By recognizing the radiographic abnormalities associated with these disorders, the radiologist can play a critical role in facilitating timely testing, diagnosis, and intervention. With the aim of improving radiologists’ recognition of possible MPS disease across a broad spectrum of presentations and beyond the classic phenotypes, we reviewed the radiographs and clinical information of 8 patients who were initially diagnosed with or suspected of having other skeletal disorders, and discuss the radiographic findings that might generate suspicion of MPS.

## Materials and methods

We reviewed the medical records of 5 MPS IVA and 3 MPS VI patients with delayed diagnosis. Radiographs were retrospectively reviewed by a pediatric radiologist (R.S. Lachman) with special expertise in skeletal dysplasias. In addition, confirmed cases of MPS in the radiographic database of the International Skeletal Dysplasia Registry were analyzed to determine the referring and actual diagnoses.

## Results

### Case 1

Patient 1, a Caucasian female, presented with mid-thoracic back pain and scoliosis at age 9 years. Her height was 131.2 cm (25th percentile) and her weight was 25.3 kg (10th percentile). Radiographs were interpreted as revealing L1 hemivertebra, generalized platyspondyly, and mild epiphyseal dysplasia. Cardiac abnormalities (mitral and triscuspid prolapse), joint laxity, and a barrel-shaped chest were also noted. Urinary GAG excretion was normal and a diagnosis of spondylo-epiphyseal dysplasia tarda (SEDT) was made. Kyphoscoliosis continued to progress. By age 21 years, she had severe bilateral hip pain; her height at this time was 151 cm (<5th percentile). Between ages 23 and 24 years, both hips were replaced. Corneal dystrophy (pre-Descemet’s dystrophy) was noted at age 25. Vocal cord nodules were removed at age 27. The patient returned to genetics at age 29 to discuss the potential risks to future offspring. A repeat urinary GAG screen did not reveal abnormalities. However, a review and discussion of this case with a metabolic specialist prompted enzymatic activity testing for and subsequent diagnosis of MPS IVA.

**Fig. 1:**
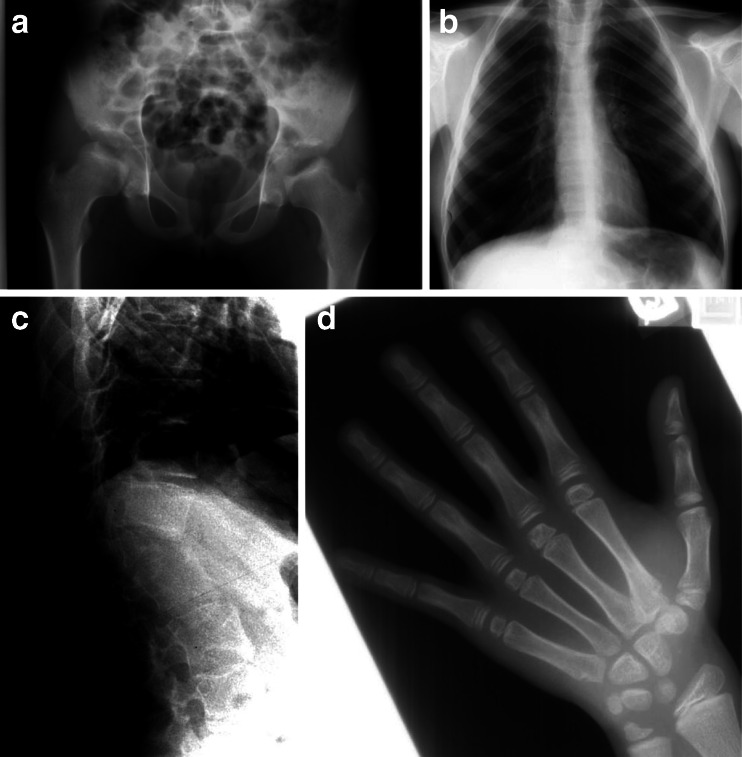
Radiographs of patient 1, female, Morquio A syndrome (MPS IVA). **a** Antero-posterior (AP) pelvis and hips, age 17 years: hypoplastic dense capital femoral epiphyses (CFE). **b** AP chest, age 17 years: normal. **c** Lateral spine, age 17 years: hypoplastic L1 vertebral body producing a significant gibbus. **d** AP hand and wrist, age 17 years: normal

Figure 1 shows radiographs of this patient obtained in late adolescence. Review of these radiographs reveals a hypoplastic L1 vertebral body (no apparent hemivertebra formation) resulting in a mild gibbus and scoliosis. There is no evidence of platyspondyly. The ribs are normal in size and contour, but down-tilted resulting in a narrow thorax. There are dysplastic capital femoral epiphyses (CFE) with normally formed iliac wings. Knee epiphyses and all other epiphyses are normal. Hand/wrist films are normal. None of these findings is characteristic of dysostosis multiplex to suggest MPS disease; however, a form of multiple epiphyseal dysplasia (MED) is suggested by this radiographic survey.

The final diagnosis was MPS IVA.

### Case 2

Patient 2, a Caucasian girl, was noted to have an abnormal gait during a routine pediatric examination at age 4. Her height and weight at age 5 years were 109 cm (9th percentile) and 20 kg (52nd percentile) respectively. At age 6, the patient was admitted to a pediatric hospital for diagnostic evaluation due to gait abnormalities, weakness, and motor developmental delay. An MRI of the hips was interpreted as normal and excluded Legg–Calvé–Perthes disease (Perthes disease). Clinical evaluations including echocardiography were normal. At age 7 years she was seen by an orthopedic surgeon. Radiographs and an MRI of the spine showed “protrusion of L2,” while re-evaluation of the hip MRI and a hip radiograph revealed bilateral flattening of the epiphyses of the femoral heads. She was diagnosed with MED. A second orthopedic surgeon suspected SED because of some involvement of the spine and a genetics evaluation was recommended. Suspicion of MPS IVA prompted diagnostic testing for lysosomal storage disorders. Urinary GAG excretion was normal, but reduced GALNS activity led to a diagnosis of MPS IVA at age 8. Her height at diagnosis was 119.5 cm (3rd percentile).

**Fig. 2:**
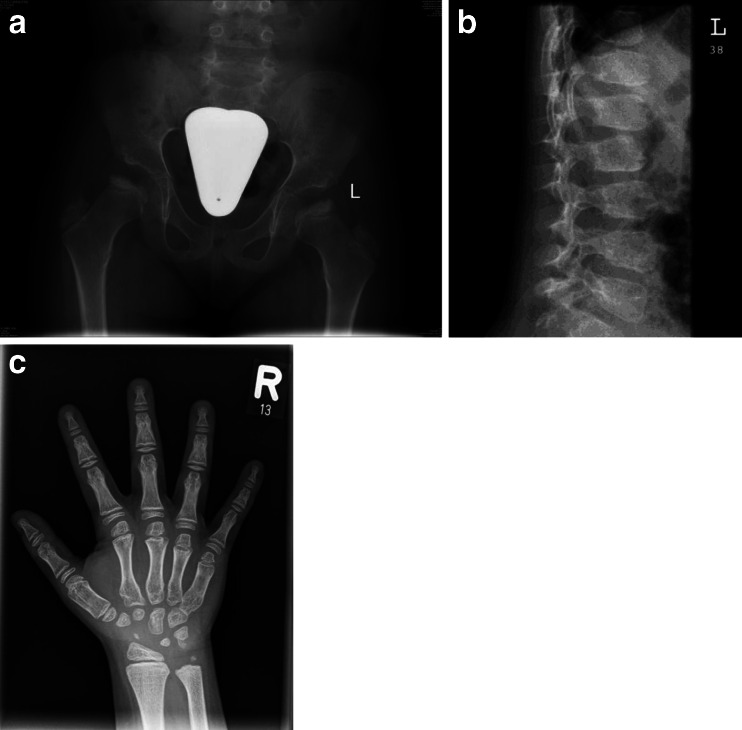
Radiographs of patient 2, female, MPS IVA. **a** AP pelvis and hips, age 7 years: normal pelvis; bilateral dense/dysplastic CFE. **b** Lateral lumbar spine, age 6 years: slightly abnormal with mild residua of the middle beaking of several vertebrae (T11, T12) and a slightly superior notched L1. **c** AP right hand and wrist, age 8 years: mild generalized brachydactyly; wide growth plates and hypoplastic carpal centers; no significant proximal pointing, but poor proximal metacarpal formation for age

Radiographic review of the plain films at ages 6–8 years (Fig. 2) reveals normal iliac wings and dense dysplastic CFE. The remaining epiphyseal centers, including the knees, are all normal. The hand and wrist film suggests mild generalized brachydactyly, mildly widened phalangeal growth plates, and poor formation of the proximal metacarpals, somewhat like proximal pointing (these changes are subtle). The carpal bones are small and dysplastic with ossification delay. The lateral spine at age 6 years reveals several vertebrae with residual mild middle beaking (T11 and T12) and one superior notched vertebra (L1). The hand and spine findings suggest dysostosis multiplex. The bilateral dysplastic CFE, mild brachydactyly, and dysplastic carpal centers suggest the possibility of some form of MED.

The final diagnosis was MPS IVA.

### Case 3

Patient 3, a Korean male, first presented with hip pain at age 11 years, and bilateral Perthes-like disease was suspected. Aggravated hip pain led him to see an orthopedic surgeon at age 27 years. He is of average stature for an adult Korean male, with a height of 169 cm and a weight of 74 kg. Radiographic abnormalities of the spine, pelvis/hips, and knees led to suspected X-linked SEDT, but this was not confirmed by mutation analysis of* SEDL* (*TRAPPC2*). Molecular testing for SEDC (*COL2A1*) and MED (*COMP*,* MATN3*,* COL9A1-3*) did not reveal any deleterious mutations either. Whole exome sequencing identified two *GALNS* mutations, c.317A>G (p.N106S) and c.553delG (p.E185Rfx14), which were confirmed by Sanger sequencing. These mutations have not been previously reported among individuals with MPS IVA or any other disease. The patient is a compound heterozygote for these two mutations. Reduced GALNS activity confirmed a diagnosis of MPS IVA.

**Fig. 3:**
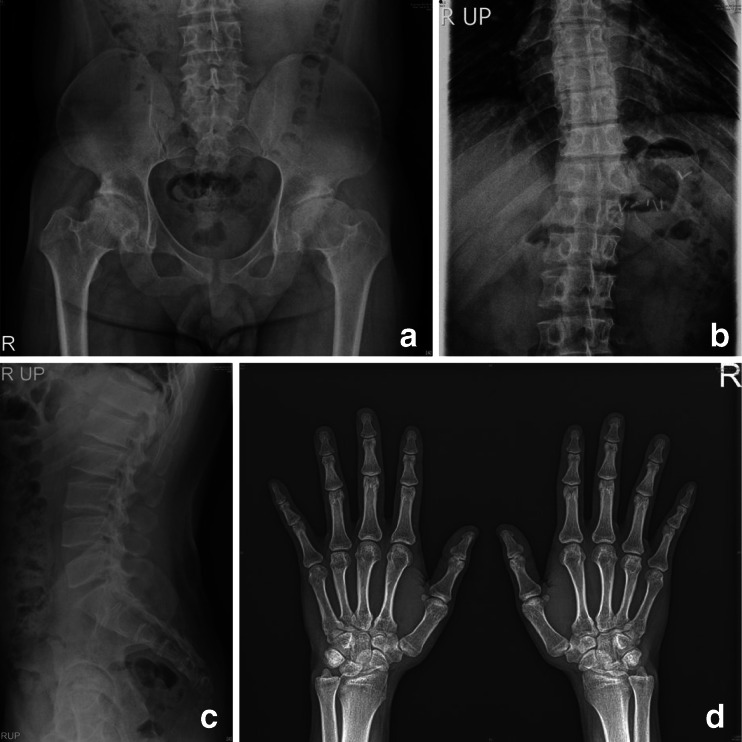
Radiographs of patient 3, male, MPS IVA. **a** AP pelvis and hips, age 27 years: mildly dysplastic CFE with joint space narrowing; sclerosis (degenerative arthrosis). **b** AP spine and lower ribs, age 27 years: scoliosis; normal paddle-shaped ribs (T9–T12). **c** Lateral spine, age 27 years: several superior humped vertebral bodies in the lumbar spine. **d** AP hands and wrists, age 27 years: normal

Radiographic review of this patient (Fig. 3) at age 27 shows kyphoscoliosis and mild platyspondyly with several superior/inferior humped vertebral bodies (L1–L3). The pelvis/hip film reveals normal iliac wings and dysplastic femoral heads with joint space narrowing and acetabular sclerosis (secondary degenerative arthrosis). The hand and wrist films appear normal. The films of the hips and spine do indeed suggest the diagnosis of SEDT. There are no findings radiographically that suggest any form of MPS. The radiographs also suggest MED as a possible diagnosis.

The final diagnosis was MPS IVA.

### Case 4

Patient 4, a 2 ½ -year-old Caucasian girl with “congenital kyphosis”, was seen by orthopedics because of parental concerns about prominent hardware, a waddling gait, and reduced mobility. A genetics evaluation at age 3½ years revealed a height of 96.5 cm (29th percentile). Proximal femoral epiphyseal flattening was detected on radiographs and a type II collagenopathy was considered; however, the mutation analysis was not consistent with a type II collagenopathy. At age 9, her height was 116.5 cm (< 3rd percentile). Mitral valve thickening, mild tricuspid insufficiency, and restrictive lung disease were observed. Slightly elevated urinary GAG levels prompted enzyme activity analysis. She was diagnosed with MPS IVA based on reduced GALNS activity. She is a compound heterozygote for two known *GALNS* missense mutations, c.776G>A (p.R259Q) and c.901G>T (p.G301C) [18].^1^

**Fig. 4:**
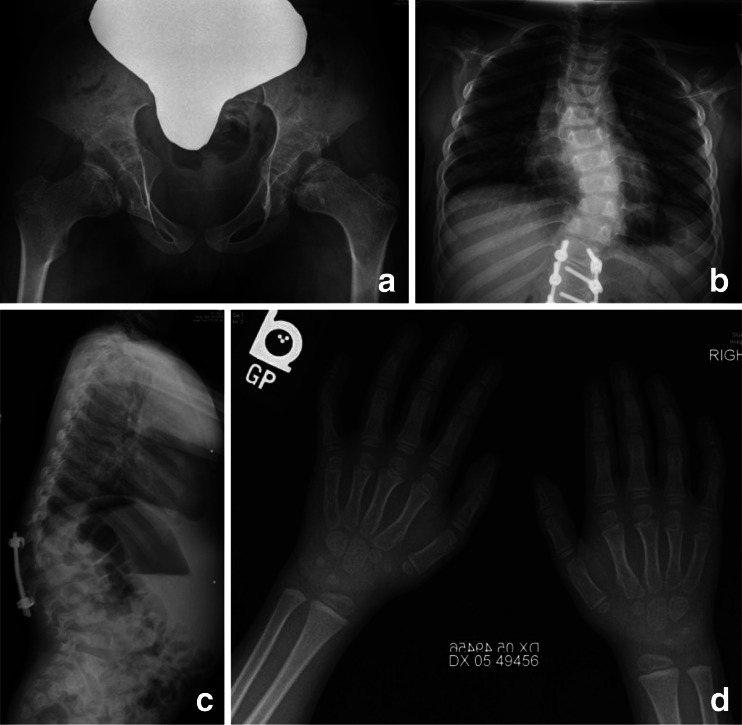
Radiographs of patient 4, female, MPS IVA. **a** AP pelvis and hips, age 9 years: dysplastic CFE; mildly tapered lower iliac wings merging into the acetabular roofs. **b** AP spine and ribs, age 9 years: severe scoliosis with hardware; normal ribs. **c** Lateral spine, age 2½ years: gibbus; hypoplastic T12; middle beaked L1. **d** AP hands and wrists, age 6 years: relatively normal, but carpal ossification delay

Review of the radiographs of this patient (Fig. 4) shows mild but significant changes of dysostosis multiplex with dysplastic CFE. Spine films at age 2½ years reveal a gibbus and scoliosis. There is a hypoplastic T12 and a middle beaked L1. On the pelvis/hip film at age 9 years, mild inferior tapering of the iliac wings is observed. Bilateral dysplastic CFE are again seen (confirmed earlier by MRI and hip arthrography), with otherwise normal epiphyseal centers elsewhere. The hands are normal aside from nonspecific carpal ossification delay. Overall, there are significant findings of dysostosis multiplex including dysplastic changes of the CFE similar to those observed in the other cases.

The final diagnosis was MPS IVA.

### Case 5

Patient 5 is a physically active Caucasian boy who started limping at age 4 years and started experiencing knee pain at age 5 years. He was diagnosed with bilateral Perthes disease at age 7. He presented to the skeletal dysplasia clinic at age 9 years, when his height was 125.2 cm (5.5th percentile) and his weight was 27.1 kg (30th percentile). Mild scoliosis was the only abnormality noted on physical examination. Two urinary GAG screens were normal. SEDC, Stickler syndrome, and X-linked SEDT were considered, but not confirmed by molecular testing. After 1 year, he returned to the clinic with a developing chest wall deformity and increasing hip pain. Reduced GALNS activity led to a diagnosis of MPS IVA. One copy of the c.901G>T (p.G301C) mutation on the *GALNS* gene was identified [18].^2^

**Fig. 5:**
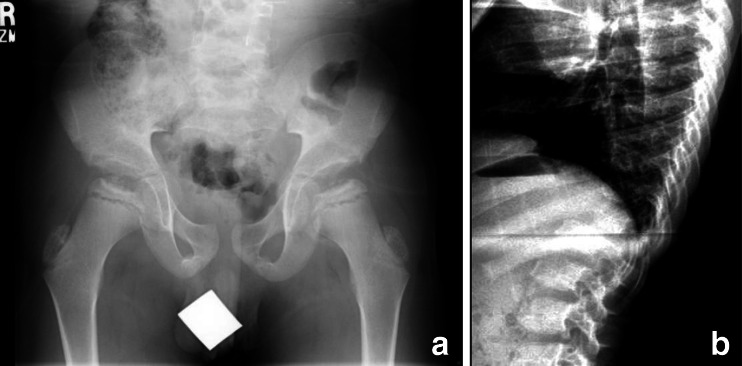
Radiographs of patient 5, male, MPS IVA. **a** AP pelvis and hips, age 7 years: dysplastic superiorly eroded CFE. **b** Lateral spine, age 9 years: kyphosis; mildly hypoplastic T11 and T12 and posterior scalloping

Retrospective evaluation of the radiographs of this patient (Fig. 5) reveals no specific evidence of dysostosis multiplex. The pelvic films reveal normal ilia, with rather poor acetabular roof formation. There are dysplastic CFEs; all other epiphyses appear quite normal. The spine radiographs show several mildly hypoplastic vertebral bodies (T11 and T12) and posterior scalloping in the thoracolumbar junctional region. Some form of either MED or SED are suggested by these films.

The final diagnosis was MPS IVA.

### Case 6

Patient 6, a Caucasian girl, was referred to orthopedics at age 6 years because of mildly short stature, an abnormal gait, and toe-walking. Her height at the time was 110 cm (10th percentile). At age 7, a diagnosis of spondylo-epiphyseal dysplasia congenita (SEDC) was established based on apparent radiographic abnormalities of the pelvis and spine. Snoring and a history of frequent ear infections prompted referral to an ear/nose/throat (ENT) specialist, and she underwent tonsillectomy and adenoidectomy at age 7. She was diagnosed with hearing loss and chronic otitis media at age 9. During recurrence risk counseling for SEDC, a geneticist clinically suspected MPS based on a photograph of the patient (coarse facial features). Enzyme activity testing at age 10 established a diagnosis of MPS VI.

**Fig. 6:**
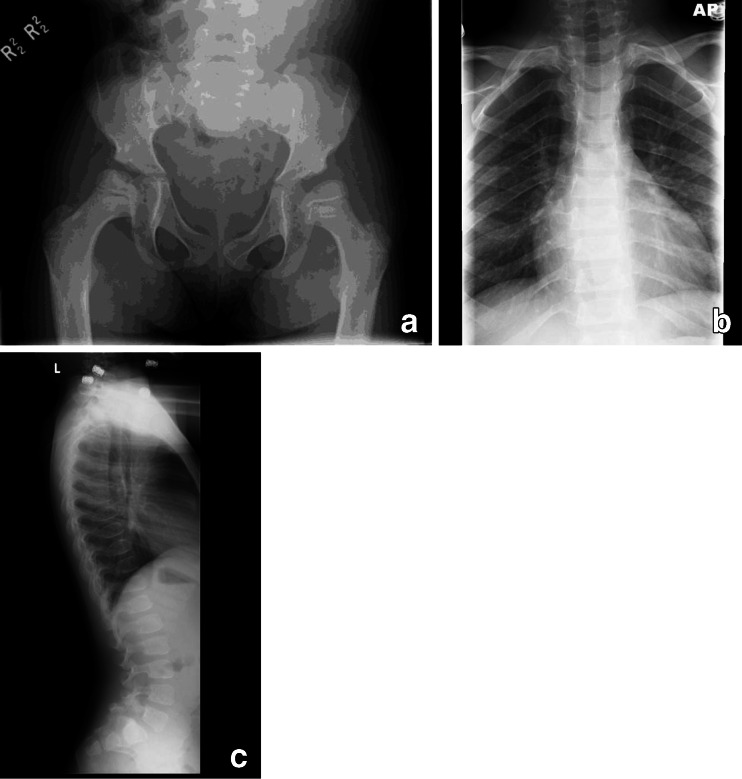
Radiographs of patient 6, female, Maroteaux-Lamy syndrome (MPS VI). **a** AP pelvis and hips, age 8 years: relatively normal pelvis; CFE are a little dysplastic with asymmetrical involvement; superior flattening, but erosive notching on the right. **b** AP spine and ribs, age 8 years: mild scoliosis (nonspecific finding); spine otherwise normal; ribs are normal without thickening/paddle shaping; clavicles are normal. **c** Lateral spine, age 8 years: mildly superiorly notched T9

Radiographs of this patient taken at age 8 were reviewed (Fig. 6). The characteristic changes of dysostosis multiplex are not evident in this patient. The asymmetrical and mildly dysplastic changes of the CFE appear to mimic MED. The lateral thoracic and lumbar spine film shows a single mildly superior notched vertebra (T9) in the lower thoracic spine (a subtle finding), which is not diagnostic of dysostosis multiplex and can be seen with hypotonia, etc. The mild scoliosis alone does not constitute sufficient spinal involvement for any well-described type of SED.

The final diagnosis was MPS VI.

### Case 7

Patient 7 is a girl of Asian origin who was seen by orthopedics at 9 years of age because of short stature. Her radiographs were interpreted as showing bilateral acetabular dysplasia with coxa magna, deformities of the femoral heads, and epiphyseal and metaphyseal abnormalities. Corneal clouding was noted, therefore the possibility of MPS was raised and the patient was referred to genetics. Physical examination revealed a height of 117.5 cm (<5th percentile), weight of 22.0 kg (5th percentile), and head circumference of 54.5 cm (+1 SD). Some limitation of range of motion of the shoulder was noted. A second review of her radiographs led to an initial impression of MED and referral to a skeletal dysplasia clinic. Bilateral corneal opacities were observed upon ophthalmological examination. At age 10 years, a heart murmur was detected. Subsequent cardiology assessments revealed mitral valve thickening, mitral valve prolapse, mild mitral regurgitation, and mild aortic insufficiency. At a follow-up genetics visit, mild valgus deformity of the knees, a waddling gait, lordosis of the spine, and a broad flat nasal bridge were noted. Review of the patient’s history and symptoms led to suspicion of an MPS disorder and prompted diagnostic testing for lysosomal storage disorders and a full skeletal survey. MPS VI was diagnosed based on low ARSB activity in leukocytes.

**Fig. 7:**
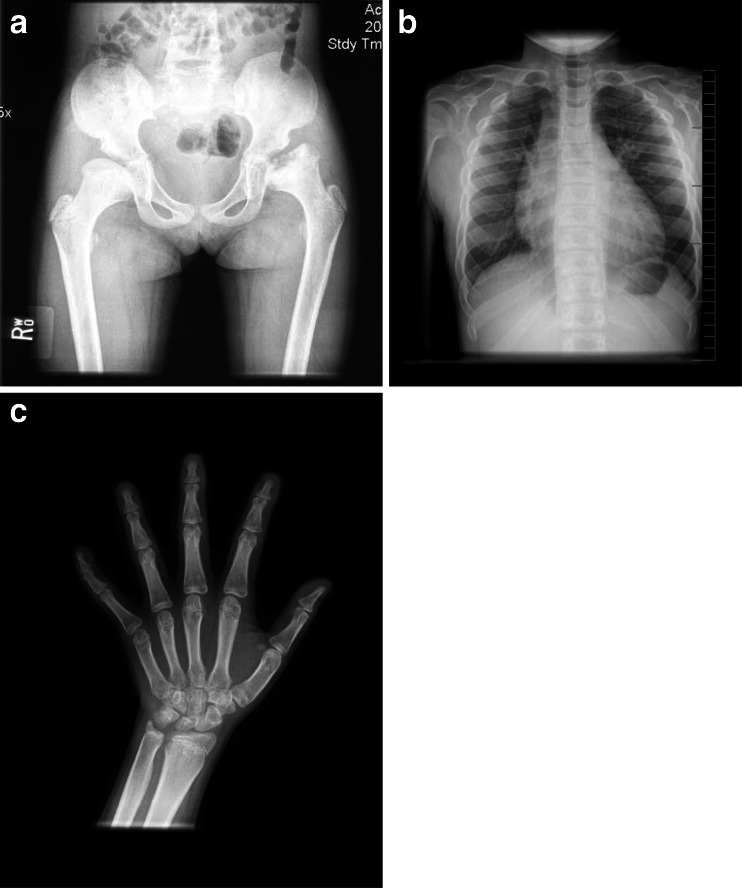
Radiographs of patient 7, female, MPS VI. **a** AP pelvis and hips, age 11 years: slightly abnormal pelvis with mild residua of inferior ileal tapering; bilateral CFE dysplasia with flattened epiphysis on the right and flat erosive changes on the left. **b** AP chest, age 11 years: the ribs are thick, but not paddle-shaped; clavicles are also thick. **c** AP hand and wrist, age 11 years: normal; no proximal pointing or brachydactyly; normal size/shape of carpal bones

Figure 7 shows the chest and pelvis/hip radiographs of this patient obtained at age 11 years (most recent modified skeletal survey). A review of these radiographs reveals thick ribs and clavicles, which are characteristic of dysostosis multiplex. The pelvis film shows mild residua of lower ileal tapering. The CFE changes are MED-like. Overall, there are some mild suggested features of dysostosis multiplex.

The final diagnosis was MPS VI.

### Case 8

Patient 8, a Caucasian boy, was diagnosed with bilateral Perthes-like disease, but was referred to genetics at age 10 years for possible skeletal dysplasia. On physical examination his height was 143 cm (65th percentile), his weight was 43.5 kg (92th percentile), and head circumference was 56.2 cm (95th percentile). He was noted to have mild scoliosis, a protuberant abdomen, thickened gums, and hyperreflexia at his knees and ankles. Radiographs were interpreted to reveal symmetrical epiphyseal abnormalities of the proximal femurs and irregularities of one vertebral endplate. His urinary GAG screen was normal; however, a trace amount of dermatan sulfate was observed. Molecular testing of the *COL2A1* gene and the *COL11A2* gene did not reveal any deleterious mutations that would diagnose SEDC and Stickler dysplasia respectively. At age 12 years, the patient returned to clinic with continuing hip pain. Repeat screening for MPS revealed mildly elevated urinary GAG excretion as well as trace levels of dermatan sulfate, prompting enzyme activity analysis for MPS VI. Low ARSB activity and subsequent molecular testing confirmed a diagnosis of MPS VI. This patient is homozygous for the c.629A>G (p.Y210C) missense mutation in the *ARSB* gene [18].^3^

**Fig. 8:**
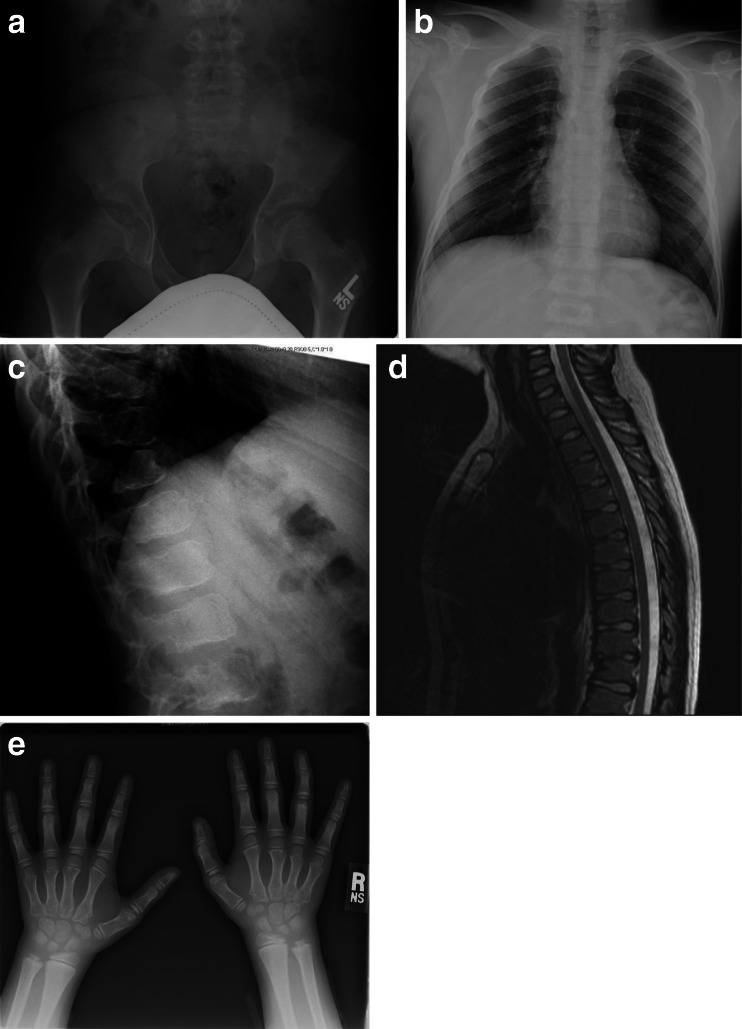
Radiographs and MR image of patient 8, male, MPS VI. **a** AP pelvis and hips, age 12 years: hypoplastic CFE. **b** AP chest, age 12 years: normal. **c** Lateral spine, age 12 years: superior notched T12 vertebral body. **d** MRI lateral spine, age 12 years: anterior/superior vertebral body irregularity/notching with intact region of cartilaginous anlage. **e** AP hands and wrists, age 10 years: normal

On review, radiographs of this patient (Fig. 8) reveal hypoplastic CFE and a single superiorly notched T12 vertebral body (nonspecific finding). No other skeletal abnormalities suggesting dysostosis multiplex are recognized.

The final diagnosis was MPS VI.

### Summary

Taken together, all of these cases demonstrate that MPS disease may often be radiographically confused with other skeletal dysplasias/entities, in particular MED, SED, and bilateral Perthes-like disease.

### International Skeletal Dysplasia Registry database search

A search in the International Skeletal Dysplasia Registry reveals that of the total 151 cases of confirmed dysostosis multiplex submitted for evaluation, approximately 75 % of referring physicians did not recognize the likelihood that their case clinically/radiologically represented a form of MPS disease. MED, SEDC, Dyggve–Melchior–Clausen dysplasia including Smith–McCort syndrome, and pseudo-achondroplasia were among the most common referring diagnoses.

## Discussion

Diagnosing MPS IVA or VI can be a challenging and protracted process, as illustrated by the cases described here. An accurate diagnosis typically requires recognition of specific clinical and/or radiographic signs and symptoms combined with laboratory confirmation, which often involves multidisciplinary input. Delayed or incorrect diagnosis can result from a number of factors including the incorrect interpretation of radiographs, lack of disease awareness, wide phenotypic variability, phenotypic overlap with other disorders, and limitations of urinary GAG screening. The radiologist can play a critical role in ensuring that an accurate diagnosis is reached expeditiously by raising suspicion of an MPS disorder if dysostosis multiplex changes are evident. However, our retrospective study reveals a significant finding: MPS disease can radiographically manifest as an apparent type of MED with isolated dysplastic CFE (MED Ribbing type) with very few, if any, additional findings suggestive of classical dysostosis multiplex. MED Ribbing type is characterized by epiphyseal ossification delay and dysplastic-appearing epiphyses, usually by definition confined to the hips [19].

As our case series illustrates, musculoskeletal abnormalities, including growth delay, abnormal gait and hip pain, are among the first clinical symptoms to appear and typically prompt a patient or the family to seek medical attention. Dysostosis multiplex, associated with the findings summarized in Table 1, is highly suggestive of the diagnosis of MPS [11]. Only a few patients in our series presented with findings of dysostosis multiplex, and many of these features were subtle. The dysplastic MED-like changes of the CFE observed in our patients, although seen at times in MPS, have not previously been described as a diagnostic feature of any form of MPS disease. This finding may be useful in identifying possible nonclassic MPS phenotypes. Our retrospective study also suggests that MPS IVA and VI might be frequently misdiagnosed as SED based on incorrect radiographic interpretation; therefore, it is important to note that a diagnosis of SED based solely on scoliosis, kyphosis, and/or a single superior notched vertebra without the presence of either platyspondyly or superior/inferior humping of vertebral bodies is not consistent with any known form of SED. This case series suggests that MPS should be considered in the differential diagnosis of MED, some forms of SED, and bilateral Perthes-like disease, including Meyer’s dysplasia [20].

**Table 1:** The manifestations of dysostosis multiplex

Skeletal region	Dysostosis multiplex manifestations
Skull	*Abnormal J-shaped sella turcica* Thickened diploic space
Thorax	Short, thick clavicles *Paddle-/oar-shaped ribs*
Spine	*Several superiorly notched (inferiorly beaked) vertebral bodies* Posterior scalloping of vertebrae *Middle beaked vertebral bodies (common in MPS IVA)*
Pelvis/hips	Rounded iliac wings *Inferior tapered ilia*
Long bones	Mildly hypoplastic epiphyses (often generalized) Hypoplastic, dysplastic, or fragmented CFE Proximal humeral notching Long narrow femoral necks Hypoplastic distal ulnae Thick short diaphyses
Hands	*Proximally pointed metacarpals* *Short broad metacarpals with thin cortices* Irregular/hypoplastic carpal bones
Feet	Tarsal bones with irregular contours

Entities in italics are classical diagnostic findings of dysostosis multiplex

*MPS IVA* mucopolysaccharidosis IVA (or Morquio A syndrome),* CFE* capital femoral epiphysis

Conducting a full skeletal survey is mandatory if an MPS or other skeletal dysplasia is suspected; imaging only one or a few regions is likely to result in missed findings and may lead to a delayed or incorrect diagnosis. It is also important to note that some changes of dysostosis multiplex, such as proximal pointing of the metacarpals, vertebral body changes, and lower ileal changes, may become impossible to detect after the time of epiphyseal plate fusion. For patients who may not present with apparent clinical symptoms until late childhood or adulthood, review of early or pre-pubertal radiographs can be quite helpful if they are available.

If an MPS disorder or other skeletal dysplasia is suspected, a comprehensive examination that includes clinical findings must be conducted, which requires collaboration between the radiologist, referring clinician, and the clinical geneticist. Extra-skeletal manifestations, including corneal clouding, specific cardiac abnormalities, and facial dysmorphology, can provide vital clues for achieving a correct diagnosis of MPS, but are often overlooked or missed due to a lack of disease awareness, as demonstrated by several of our cases.

Diagnostic enzyme testing is required for the definitive diagnosis of MPS IVA or VI [4, 5]. Urinary GAG analysis alone is unreliable and can be misleading, as illustrated by several of the cases. Molecular analysis can be useful in diagnosing or confirming a diagnosis of an MPS disorder. Unless two known pathogenic gene mutations have been identified on separate alleles, enzyme activity testing should always be used to confirm a diagnosis of MPS IVA or VI. If molecular testing is not performed to confirm enzyme activity results, it is important to measure the activity of a second sulfatase enzyme to exclude multiple sulfatase deficiency [4, 5]; additionally, for suspected MPS IVA, it is highly recommended to also measure β-galactosidase activity to rule out MPS IVB [4]. Accurate diagnosis requires corroboration of complete radiographic and clinical information with the laboratory findings. Once diagnosed, MPS IVA and VI patients should receive multidisciplinary care and can be considered for ERT or other therapeutic intervention if such treatment modalities are available.

## Conclusions

While recognition of dysostosis multiplex changes can facilitate the timely diagnosis of MPS disease, our study indicates that MPS IVA and VI can manifest radiographically as MED with isolated dysplastic CFE (MED Ribbing type), with very few, if any, additional findings to suggest dysostosis multiplex. The cases presented here illustrate the wide phenotypic variability of these disorders, underscore the importance of considering MPS in the differential diagnosis of certain skeletal dysplasias/disorders, particularly MED, SED, and bilateral Perthes-like disease, and emphasize the need to combine radiographic, clinical, biochemical and molecular findings to achieve an accurate diagnosis.

## Footnotes

1 Previously published in Mendelsohn et al. [18]

2 Previously published in Mendelsohn et al. [18]

3 Previously published in Mendelsohn et al. [18]
